# Long-Term Outcomes of Retinal Detachment in Phakic Eyes After Implantation of Implantable Collamer Lens V4c for High Myopia Correction

**DOI:** 10.3389/fmed.2020.582633

**Published:** 2020-12-15

**Authors:** Weiwei Xu, Zhou Song, Yifei Huang, Ye Tao, Junqing Wang, Liqiang Wang, Zhaohui Li

**Affiliations:** ^1^Department of Ophthalmology, Chinese People's Liberation Army General Hospital, Beijing, China; ^2^Department of General Surgery, Chinese People's Liberation Army General Hospital, Beijing, China; ^3^Department of Ophthalmology, Henan Provincial People's Hosptial, Zhengzhou, China

**Keywords:** myopia, phakic intraocular lens, V4c, rhegmatogenous retinal detachment, incidence, morbidity

## Abstract

**Aim:** To estimate whether implantable collamer lens (V4c ICL) implantation increases the risk of retinal detachment in high myopia comparing with myopes with Rigid Gas-Permeable (RGP) correction.

**Methods:** This prospective study was comprised of an ICL group (704 eyes) and an RGP group (628 eyes). Patients were enrolled according to the inclusion criteria and exclusion criteria, then divided into the ICL group and RGP group. Patients in the ICL and RGP groups received V4c ICL implantation and RGP fitting respectively. Retinal details, spherical equivalent refraction (SE), uncorrected distance visual acuity (UDVA), corrected distance vision acutivity (CDVA), axis length (AL), anterior chamber depth (ACD) and other relevant parameters were recorded at different time points. Rhegmatogenous retinal detachment (RRD) morbidity and incidence, RRD morphology and relevant parameters were analyzed.

**Results:** All enrolled patients were followed for 3–6 years. Patients characteristics before the refractive procedure did not show a statistical difference. At the end of the follow up, all the RD cases were RRD. The RRD morbidity of the ICL group and RGP group was 1.99% (14 eyes) and 0.96% (6 eyes) respectively, which did not show statistical difference (*P* = 0.12). During the first year after refractive procedure, the RRD incidence of the ICL group was 0.85% (6/704), while this number of the RGP group was 0.16% (1/628). It did not show statistical difference (*P* = 0.08).

**Conclusion:** Compared with RGP fitting, V4c ICL implantation for high myopia correction does not add RRD risk in the long term. V4c ICL implantation is a safe method for high myopia correction.

## Introduction

Myopia is one of the most common ametropic diseases (1). In contrast with emmetropic eyes, patients with high myopia have a higher incidence of retinal detachment (RD) (2). Common treatment of high-degree myopia includes clear lens extraction with intraocular lens (IOL) implantation, Phakic intraocular lens (pIOL) implantation, corneal refractive surgery, and Rigid Gas-Permeable (RGP) lenses or spectacles. Compared with corneal refractive surgery, pIOLs have the advantage of treating a much larger range of myopic and hyperopic refractive errors. Thus, pIOLs have become a popular option for high myopia. Implantable pIOLs come in two broad varieties: Anterior Chamber Intraocular Lenses (ACIOLs) and Posterior Chamber Intraocular Lenses (PCIOLs). In PCIOLs implantation, the lens is implanted between the iris and the crystalline lens, resulting in good correction of myopia (3). Posterior Chamber Intraocular Lenses offers several advantages for correction of high-degree myopia: reversibility, a greater amount of correction, a minimally invasive, precise predictable, preservation of accommodation and corneal endothelia protection. In recent years, ACIOLs implantation has gradually been replaced by PCIOL implantation (3). Its early generations had some defects, such as: poor predictability, and higher risk for developing glaucoma and cataract. To rectify this, the 4th generation implantable collamer lens (V4 ICL) was designed to provide more space between ICL and crystalline lens and prevent cataract formation (4). As a result, pre-operative peripheral iridectomy is needed, to prevent pupillary block. However, peripheral iridectomy increased the risk of intraocular inflammatory reaction and hyphema. This lengthens the entire treatment period, since patients who undergo pre-operative peripheral iridectomy often have to wait for at least 2 weeks before V4 ICL implantation. To solve these problems, “CentraFLOW Technology” was introduced in the design. That is V4c ICL. It is a single piece posterior chamber phakic refractive intraocular lens designed with a central hole. This allows aqueous humor could flow between the anterior and posterior chamber to maintain the normal physiology of the anterior segment of the ocular, eliminating the need of pre-operative peripheral iridectomy. Recent researches have compared V4 and V4c ICL implantation, and confirmed that there was no significant difference in visual quality (5, 6).

In our center, V4c ICL has been used widely for correction of high myopia. Some patients developed RD with monocular or binocular implantation. Because high myopia leads to pre-mature vitreous liquefaction, posterior vitreous detachment, and an increased prevalence of lattice degeneration, it is a significant risk factor for rhegmatogenous retinal detachment (RRD) (2, 7). It is not clear whether V4c ICL implantation increases the risk of RRD. Therefore, the present study was designed to determine whether V4c ICL implantation increases the risk of RRD in high myopia. RGP correction was chosen as a control.

## Materials and Methods

### Participants

This is a prospective, comparative cohort study. This study followed the Declaration of Helsinki and was approved by Chinese PLA General Hospital Medical Ethics Committee. All participants were well-informed about advantages and disadvantages of V4c ICL implantation and RGP lenses fitting for the correction of high myopia. All participants signed the informed consent form, including 352 patients (704 eyes) underwent V4c ICL implantation and 315 patients (628 eyes) received RGP fitting. All the cases were treated at Chinese PLA General Hospital between March 1, 2014, and March 1, 2017. The inclusion and exclusion criteria were as follows.

Inclusion criteria:

1. 18–50 years old, with a stable refraction for more than 2 years;
2. Not suitable for corneal laser surgery or patients refuse corneal laser surgery;
3. A pupil diameter (dim light) ≤ 6 mm;
4. Corneal endothelial cells: ≥2,000/mm^2^;
5. Corneal diameter range: 10.6–12.5 mm;
6. Anterior chamber depth (excluding central cornea thickness) ≥2.8 mm;
7. Myopia diopter −5D to −18.00D; astigmatism −0.5D to −5.00D.

Exclusion criteria:

1. Prior corneal laser surgery;
2. Have or had any ophthalmic diseases other than refractive error;
3. Patients with systemic disease who are not suitable for intraocular surgery;
4. Patients who are unable to understand the risks of the surgery, hold unreasonable expectations or are unwilling.

Upon enrollment, we explained the advantages and risks of each therapy, making sure they were understood completely. Patients were divided into two groups (V4c ICL group or RGP group) according to their decisions. All patients enrolled made decisions by themselves and they had to pay for the procedures themselves.

### Surgery and Optometry Procedures

When the patients were enrolled, every patient had a dilated fundus examination. Retinal photocoagulation was applied in the conditions below: retinal hole without retinal detachment or subretinal fluids, lattice degeneration of retina, retinal neovascular and vitreoretinal traction. As long as the patients had these lesions, no matter the quantity of the lesion, photocoagulation should be applied. Prophylactic laser photocoagulation (argon green-laser, 100–200 mW, 300 μm, and 0.1-s retinal spots; three rows of confluent burns were made around the retinal lesions) was performed when patients had lattice degeneration, retinal tear, or retinal hole. If the patient had retinal hole combined with retinal detachment, we excluded him/her from the present research. For these patients, the following ICL implantation or RGP fitting was performed 1 month after retinal laser photocoagulation.

V4c ICL implantation was performed by the same surgeon. Oxybuprocaine 0.4% (Benoxil, Santen, Japan) was used for surface anesthesia. An incision was made at the 10 o'clock position on the right eye and 2 o'clock on the left eye using a 15°knife (Alcon, USA). Viscoelastic (DisCoVisc, Alcon, USA) was injected into the anterior chamber. Then, a 2.8 mm temporal transparent corneal incision was created. V4c ICL was inserted by a special syringe into the anterior chamber and then pushed into the posterior chamber using an aligning hook. Then the position of V4c ICL was adjusted to make sure that the optical center was midline. Finally, irrigation and aspiration were performed using BSS (Alcon, USA).

RGP contact lens was fitting according to optometry results, Pantacam, corneal topography, and axial length. RGP fitting was performed by the same optometrist.

### Parameters Recorded

When the patients were enrolled, SE, UDVA, CDVA, AL, ACD, corneal endothelium count, intraocular pressure (IOP), lattice degeneration, retinal tear(s), and retinal hole(s) were carefully examined and recorded. For patients having retinal complication, refractive procedures were performed 1 month after retinal laser treatment. Three to seven days before refractive procedure, patients received ophthalmic general and optometry special examinations. Parameters before refractive procedure were recorded at this time.

After refractive procedures (ICL implantation or RGP fitting), we requested the patients to follow-up in clinic at 1 week, 1 month, 3 months, 6 months, and 1 year intervals. After the first year, all patients followed-up annually. All the aforementioned parameters were recorded during these visits.

When RD occurred, scleral buckling (SB), extrascleral compression (EC) or pars plana vitrectomy (PPV) were performed. These surgeries were performed by the same surgeon. UDVA, CDVA, the time between refractive procedure and RD development (RD interval) were recorded at onset of RD. Patients with RD were followed at 1 week, 1, 3, 6 and 12 months after retinal detachment surgery. All these patients were re-evaluated yearly for SE, UDVA, CDVA, fundus examination, ultrasound and macular OCT examinations.

### Statistical Analysis

All statistical analyses were performed using the software Statistical Package for the Social Sciences (SPSS) Version 20.0 (SPSS, Chicago, IL, USA). A chi-square test was used to detect the difference between the two groups' ratios. Mixed-effects regression are used for double-eye data to compare the parameters between time points.

## Results

All enrolled patients were followed for 3–6 years after the initial refractive procedure. 352 patients (704 eyes) in the ICL group and 315 patients (628 eyes) in the RGP group completed the present research. At the time of the refractive procedure, there was no significant difference at age, AL, SE, and pre-operative CDVA between ICL and RGP groups. We found that patients needed prophylactic laser photocoagulation in both groups, but we did not detect statistical difference (*P* > 0.05). Patients' characteristics before the refractive procedure are summarized in Table 1. For ICL group, mean pre-operative CDVA (logMAR) was 0.09 ± 0.09, mean post-operative UDVA (logMAR) was 0.02 ± 0.04, *P* < 0.05. For RGP group, mean pre-operative CDVA (logMAR) was 0.09 ± 0.10, mean post-operative UDVA (logMAR) was 0.01 ± 0.05, *P* < 0.05.

**Table 1:** Patients' characteristics before refractive procedure.

	**ICL**	**RGP**	***P***
Age (year)	25.57 ± 4.33	25.23 ± 4.61	0.34
Sex	154M/198F	127M/188F	0.37
AL (mm)	28.18 ± 1.08	28.07 ± 1.15	0.06
SE (D)	−12.48 ± 3.22	−12.14 ± 3.45	0.06
Pre-CDVA	0.83 ± 0.16	0.85 ± 0.23	0.17
Prophylactic photocoagulation (eye)	81/704 (11.5%)	78/628 (12.4%)	0.61

This table shows patients' characteristics before refractive procedure. When the patients were enrolled, we examined all the patients' retinas. Patients with lattice degeneration, retinal tear, or retinal hole (not combined with retinal detachment) received prophylactic laser photocoagulation. Eighty-one eyes in the ICL group and 78 eyes in the RGP group received prophylactic photocoagulation. Refractive procedures were performed 1 month after retinal laser treatment. Three to seven days before the refractive procedure, patients received ophthalmic general and optometry special examinations. Parameters before refractive procedure were recorded at this time.

*Pre-CDVA, corrected distance visual acuity before refractive procedure; SE, spherical equivalent refractive accuracy; D, diopters; mm, millimeter; M, male; F, female*.

**Figure 1:**
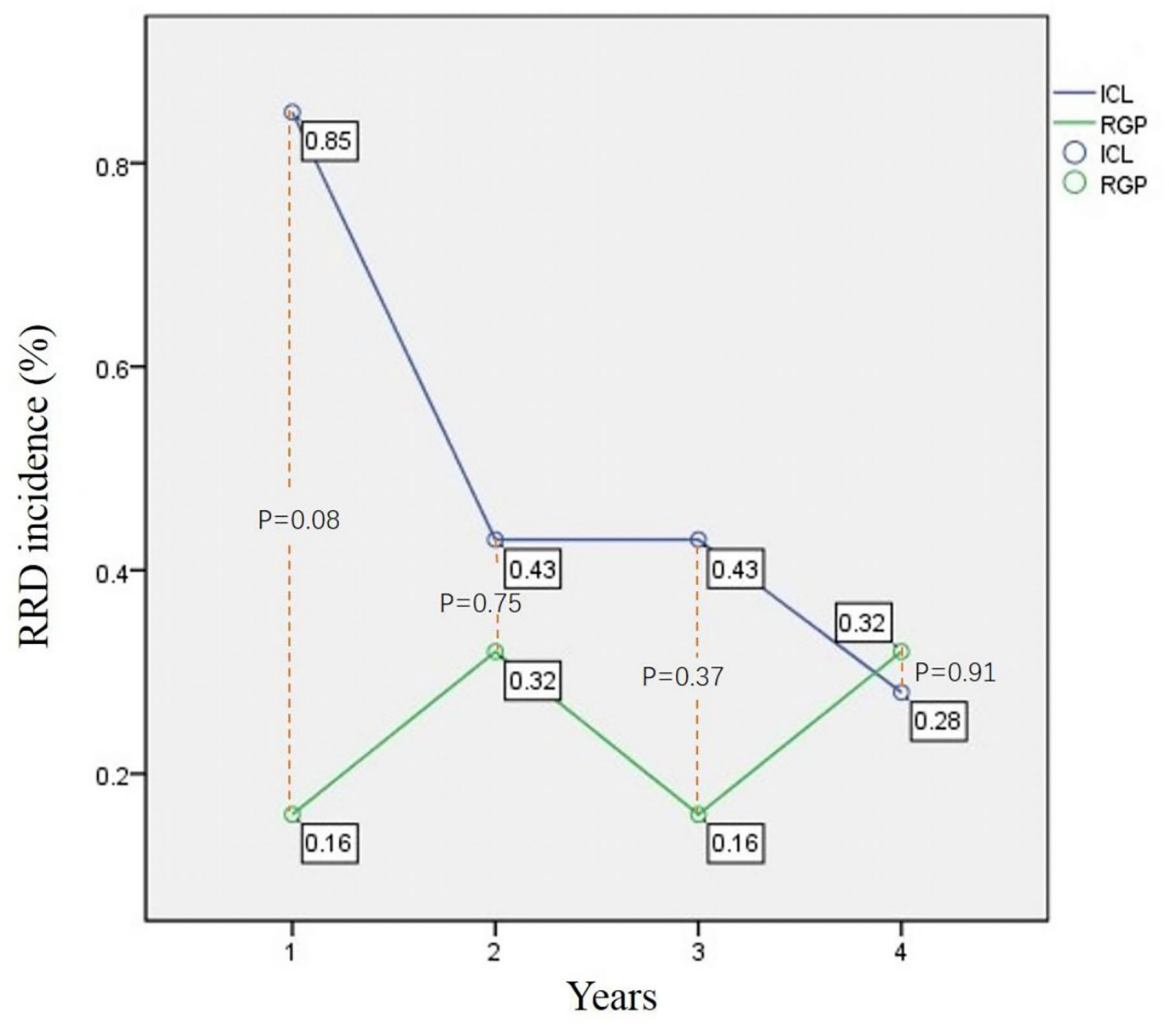
RRD incidence trend of ICL group and RGP group. X-axis is time axis. 1, the first year after refractive procedure; 2,the second year after refractive procedure; 3, the third year after refractive procedure; 4, from the beginning of the fourth year after refractive procedure to the end of follow up. This figure shows the RRD incidence trend of ICL group and RGP group in different time after the refractive procedure. The blue line is ICL group and the green line is RGP group. The ICL line is above the RGP line. However, the difference was not statistically significant.

In both groups, retinal detachment occurred in 20 eyes and all of the 20 eyes were rhegmatogenous retinal detachment. Characteristics of RRD patients are summarized in Table 2. At the end of follow-up, 14 eyes occurred RRD in ICL group and 6 eyes occurred RRD in RGP group. The morbidity of RRD in ICL and RGP was 1.99% (14 eyes) and 0.96% (6 eyes) separately. The difference did not show statistical significance, *P* = 0.12. Mean age, mean SE, mean AL of the two groups' RRD patients, the RD interval did not show significant difference. For both groups, the incidence of RRD in different years are summarized in Table 3. This result concurs with the incidence of RRD reported in previous series of posterior chamber phakic lenses (8–11). In these RRD cases, some patients had received prophylactic laser photocoagulation before refractive procedure. In ICL group, 2 of the RRD patients (2/14) had photocoagulation treatment before refractive procedure. While in RGP group, this number is one (1/6). We compared this ratio and did not detect statistical difference (*P* = 0.81). Rhegmatogenous retinal detachment incidence trends of both groups are shown in Figure 1. For ICL group, RRD incidence showed a decreased trend. In the first year, it was 0.85%, seems higher than that of other years. But it did not show statistical difference comparing with other post-operative years' incidence, *P* = 0.58. For the RGP group, the RRD incidence of different years showed a steady trend, ranging from 0.16 to 0.32%. We also compared RRD incidence in the first year after refractive procedure, between the two groups. We did not find statistical difference between the two groups (0.85 vs. 0.16%, *P* = 0.08) (Table 3).

**Table 2:** Characteristics of RRD patients at the end of follow-up.

	**ICL**	**RGP**	***P***
RRD morbidity	14/704, 1.99%	6/628, 0.96%	0.12
RD interval	19.58 ± 15.97	22.50 ± 17.27	0.72
Age	26.29 ± 4.05	29.50 ± 7.53	0.23
SE	−13.54 ± 3.42	−14.63 ± 3.96	0.54
AL	28.53 ± 1.17	28.93 ± 1.36	0.51
SEX	9M/5F	5M/1F	0.39
Break	10H/4A	5H/1A	0.57

This table shows characteristics of patients in which retinal detachment occurred. At the end of follow up, 14 eyes in the ICL group and 6 eyes in the RGP group experienced retinal detachment, and all the 20 eyes were rhegmatogenous retinal detachment.

*RRD, rhegmatogenous retinal detachment; RRD morbidity, at the end of follow-up, RRD eye number/included eye number; RD Interval, the time between refractive procedure and RD development; SE, spherical equivalent refractive; AL, axial length; M, male; F, female; Break, Morphologic features of open retinal breaks; H, horseshoe tears; A, Atrophic holes*.

**Table 3:** RRD incidence in different years after refractive procedure.

	**First**	**Second**	**Third**	**Fourth year**
	**year**	**year**	**year**	**to final point**
ICL (eyes/%)	6, 0.85%	3, 0.43%	3, 0.43%	2, 0.28%
RGP (eyes/%)	1, 0.16%	2, 0.32%	1, 0.16%	2, 0.32%
*P*-value	0.08	0.75	0.37	0.91

We compared RRD incidence between ICL and RGP groups in different years separately. There was no statistical difference in any year's result.

*Eyes, RRD eyes; First year, the first year after refractive procedure; Second year, the second year after refractive procedure; Third year, the third year after refractive procedure; Fourth year to final point, from the beginning of the fourth year after refractive procedure to the end of follow up*.

In the 14 RRD eyes of ICL group, 11 eyes had causative breaks located at peripheral retina and 3 eyes had causative break located at posterior pole. In RGP group, the break site was 4 eyes vs. 2 eyes. We compared the break site, and found no difference, *P* = 0.57. In all the 20 eyes, retinal break extended from 1 to 4 quadrants. In ICL group, 10 eyes (71.43%) were horseshoe tears and 4 eyes (28.57%) were atrophic holes, while in RGP group, this ratio was 5 eyes (83.33%) vs. 1 eye (16.67%). This ratio had no statistical significance, *P* = 0.57 (Table 2).

In ICL group, the SB or EC procedure was performed in 10 eyes (71.43%), while in RGP group, this procedure was performed in 4 eyes (66.67%). Eleven eyes underwent subretinal fluid drainage. Pars plana vitrectomy alone was performed in 4 eyes in the ICL group (28.57%) and 2 eyes in the RGP group (33.33%), with posterior breaks or large extent breaks. Air was used as a tamponade in 2 eyes and silicon oil was used in 4 eyes. Retinal laser photocoagulation was used in all the six eyes that received PPV. One eye (7.14%) of the ICL group underwent additional vitreoretinal surgery after the initial SB procedure because of proliferative vitreoretinopathy. A retinal reattachment rate was achieved in 15 of 16 eyes (93.75%) with a single procedure. The final reattachment rate was 100%. We analyzed the CDVA of the RRD patients. Before the retinal reattachment procedure, the CDVA (logMAR) of RRD patients was 0.96 ± 0.30 (ICL group) and 1.15 ± 0.37 (RGP group) respectively, *P* < 0.01. After retinal reattachment surgery, the CDVA (logMAR) of these RRD patients was 0.33 ± 0.17 (ICL group) and 0.50 ± 0.31 (RGP group) respectively, *P* < 0.01.

## Discussion

Phakic lens implantation has been widely used in recent years for the correction of high-degree myopia and offers a wider range of possible refractive correction compared with corneal refractive surgery in high myopic eyes. A new posterior chamber implantable collamer lens (V4c ICL, Staar Surgical Co.) was designed with a 360 μm central hole that allows for the natural flow of aqueous humor without the need for a peripheral iridotomy. This lens provides excellent visual performance almost equivalent to that of the conventional phakic IOL (ICL V4) (4) and may reduce the risk of anterior segment complications, such as glaucoma, anterior capsular opacification, or cataract formation (12–16).

Different authors have reported the incidence and characteristics of RRD after anterior chamber IOL implantation in phakic eyes (10, 17, 18). However, the material, design and implantation procedure of V4c ICL are quite different from those of anterior chamber IOL. To the best of our knowledge, there is currently limited literature available regarding the long-term vitreoretinal risks related to this new posterior chamber phakic IOL.

It is well-known that high myopia is an RRD risk factor; mainly because of pre-mature vitreous liquefaction, posterior vitreous detachment, and an increased prevalence of lattice degeneration (2). One study from Taiwan (19) reported high myopia with RRD was noted in 10.51% of patients. In our series, at the end of follow-up, RRD morbidity of all patients and ICL group was 1.5 and 1.99% respectively. These data were lower than the data from Taiwan. In our research, mean age of enrolled patients was about 25 years, younger than that of Chen's research. Vitreous liquefaction and posterior vitreous detachment are milder in young people. The age character supported the lower RRD incidence of our research. On the other hand, in our research, patients who had lattice degeneration, a retinal tear, or retinal hole received prophylactic laser photocoagulation. This procedure may also decrease the risk of RRD.

Our result does not support that V4c ICL implantation would increase the risk of RRD. It is necessary to analyze data from the ICL group and the control, RGP group. In the first year after refractive procedure, RRD occurred in six eyes of the ICL group, the incidence was 0.85%. This number of RGP group was one eye (0.16%). Although the difference did not show statistical significance (*P* = 0.08), the *P*-value was very close to 0.05. The more RRD cases of ICL group in the first year implies that V4c ICL implantation maybe disturbed the stability and balance of intraocular structure. One conspicuous factor is the fluctuation of IOP. Fluctuation of IOP can happen in several steps during the operation, such as making transparent corneal tunnel, injecting viscoelastics, settling the collamer lens into posterior chamber, and adjusting its location. The IOP fluctuation happened during the operation and the influence persisted for several days after the operation (20). The IOP fluctuation was delivered to the ciliary body, ora serrata, and posterior vitreous through vitreous body. This could increase the risk of posterior vitreous detachment. Both IOP fluctuation and posterior vitreous detachment exerted tractional forces on the retina, which could increase the risk of retinal shearing and RRD.

It is quite usual that the ciliary groove, or ora serrata, could be pulled when the surgeon settled all four feet of V4c ICL into posterior chamber, or adjusted the cylinder axis. These lesions will likely occur more frequently and to a greater degree when the surgeon is unskilled. These two factors maybe influence the RRD incidence of ICL group in the first year after V4c ICL implantation. However, we did not find statistical difference between the RRD incidence of ICL group and RGP group. Thus, we could not explicitly point out that V4c ICL implantation leads to RRD.

In ICL group, the incidence of RRD in the second, third and subsequent years (the time between the beginning of the fourth year and the end of follow-up) was 0.43, 0.43, and 0.28% respectively. These results showed a decreasing trend (Figure 1). This implies that the V4c ICL implantation is safe and stable in the long term. V4c ICL is a posterior chamber phakic implantable collamer lens. Its optic zone diameter is 4.9–5.8 mm, with a 360 μm central hole that allows for the natural flow of aqueous humor. ICL power calculations are performed by the manufacturer (STAAR Surgical) using a modified vertex formula. The size (length) of V4c ICL includes 12.1, 12.6, 13.2, and 13.7 mm. This size is determined by the surgeon based on the patient's WTW, ACD and other relevant parameters. Determination of the size is highly individual, and thus, a surgeon's clinical experience plays an important role in this procedure. The decreased trend of the ICL group's RRD incidence implies that V4c ICL design conforms better to the biological and anatomical structure. It also suggests that the method which we used to determinate the V4c ICL size is reasonable.

In the RGP group, the incidence of RRD in the second, third and subsequent years was 0.32, 0.16, and 0.32% respectively, which showed a steady trend (Figure 1). This suggests that RGP correction did not have significant influence on intraocular structure. Rhegmatogenous retinal detachment incidence of The ICL group was relatively higher in the first year, but the incidence of the two groups showed different trends. The RRD morbidity of the two groups did not show a significant difference at the end of follow-up (*P* = 0.12).

In the present study, the mean age of RRD cases from the ICL group was 26.29 ± 4.05 years. The types of retinal breaks were horseshoe tears and atrophic holes. Chen's (19) study reported that the incidence of RRD in high myopia patients had an obvious peak at 50–69 years of age, and a secondary peak at 20–29 years. The major type of retinal breaks were holes, or lattice degeneration with holes, in contrast to patients aged over 50 years. The clinical characteristics of RRD in the present study were consistent with Chen's (19) study, which may reveal the facts that the RRD in ICL group was due to high myopia but not V4c ICL implantation.

## Conclusion

Our results demonstrated the RRD morbidity following V4c ICL implantation and RGP correction are equivocal. V4c ICL implantation does not add RRD risk in the long term, and is safe for retinas when used for high myopia correction.

## Data Availability Statement

The original contributions generated in the study are included in the article/supplementary material, further inquiries can be directed to the corresponding authors.

## Ethics Statement

The studies involving human participants were reviewed and approved by People's Liberation Army General Hospital Ethics Committee. The patients/participants provided their written informed consent to participate in this study.

## Author Contributions

WX designed and carried out the clinical trial, collected the parameter, analyzed the data, and draft the manuscript. ZS analyzed the data and draft part of the manuscript. During interactive review process, he has made prominent contribution to data analyzation and revising the manuscript. YT analyzed some of the data and gave advice during interactive review. JW polished the manuscript. YH and ZL designed and supervised the clinical trial. LW supervised the clinical trial. All authors contributed to the article and approved the submitted version.

## Conflict of Interest

The authors declare that the research was conducted in the absence of any commercial or financial relationships that could be construed as a potential conflict of interest.
